# Advanced Solubilization of Brazilian Cerrado Byproduct Extracts Using Green Nanostructured Lipid Carriers and NaDESs for Enhanced Antioxidant Potentials

**DOI:** 10.3390/antiox14030290

**Published:** 2025-02-28

**Authors:** Victor Carlos Mello, Giovanna Oliveira de Brito, Marina Arantes Radicchi, Isadora Florêncio, Tathyana Benetis Piau, Eduardo Antonio Ferreira, Leonardo Fróes de Azevedo Chang, Ariane Pandolfo Silveira, Marina Mesquita Simões, Karen Letycia Rodrigues de Paiva, Mac-Kedson Medeiros Salviano Santos, Nicole Santana Alves, Cesar Koppe Grisolia, Sônia Nair Báo, Eliana Fortes Gris

**Affiliations:** 1 Cooil Cosmetics, Brasília 72622-401, Brazil; maradicchi.bep@gmail.com (M.A.R.); isadoraflorenciofs@gmail.com (I.F.); nicolesalves18@gmail.com (N.S.A.); leonardochang93@gmail.com (L.F.d.A.C.); pandolfo.ariane@gmail.com (A.P.S.); marinamesquita3007@gmail.com (M.M.S.); karendepaiva@gmail.com (K.L.R.d.P.); 2 Laboratory of Microscopy and Microanalysis, Department of Cell Biology, Institute of Biological Sciences, University of Brasília, Brasília 70910-900, Brazil; snbao@unb.br; 3 Graduate Program in Health Sciences and Technologies, Faculty of Health Sciences and Technologies, University of Brasilia, Centro Metropolitano, Conjunto A, Lote 01, Brasilia 72220-275, Brazil; giovanna.odebrito@gmail.com; 4 Laboratory of Genetic Toxicology, Department of Genetics and Morphology, Institute of Biological Sciences, University of Brasília, Brasília 70910-900, Brazil; tathyanabenetis@gmail.com (T.B.P.); grisolia@unb.br (C.K.G.); 5 Faculty of Health Sciences and Technologies, University of Brasilia, Centro Metropolitano, Conjunto A, Lote 01, Brasilia 72220-275, Brazil; eduardoferreira@unb.br; 6 Green Nanotechnology Group, University of Brasilia, Brasília 70910-900, Brazil; mackedson@hotmail.com

**Keywords:** nanostructured lipid carriers, natural deep eutectic solvents, *Spondias mombin*, phenolic compounds, bioactive encapsulation, sustainability, green nanotechnology, circular economy, agricultural byproducts

## Abstract

This study explores the development and characterization of lipid nanostructures (NLCs) containing natural deep eutectic solvents (NaDESs) derived from taperebá peel extract (*Spondias mombin*), a by-product rich in bioactive phenolic compounds, including ellagic acid and quercetin. The taperebá extract exhibited a high polyphenol content (2623 mg GAE/L) and notable antioxidant activity, as demonstrated by DPPH (258 mM TEAC/100 mL) and ABTS (495 mM TEAC/100 mL) assays. NLCs were developed using NaDESs to enhance the stability and bioavailability of the antioxidant compounds. Physicochemical characterization confirmed the formation of stable, nanometric, and monodispersed formulations with efficient encapsulation. Biological evaluation of the NLC-TAP-NaDES formulation demonstrated its remarkable capacity to mitigate oxidative stress in cells subjected to H_2_O_2_-induced ROS generation. Fluorescence imaging revealed a significant reduction in intracellular ROS levels in treated cells compared to untreated controls, confirming the antioxidant efficacy of the formulation. This outcome underscores the synergy between NaDESs and NLC systems in protecting and delivering phenolic compounds. This study highlights the potential of utilizing underexplored by-products, such as taperebá peels, to develop sustainable and effective antioxidant delivery systems. The NLC-TAP-NaDES platform combines nanotechnology with green chemistry principles, presenting significant implications for the treatment of oxidative stress-related conditions and broader applications in pharmaceutical and nutraceutical sciences. These findings contribute to advancing sustainable innovations in antioxidant therapies, leveraging the dual benefits of bioeconomy and high-performance nanomaterials.

## 1. Introduction

The Brazilian Cerrado, a vast and ecologically rich tropical savanna, is globally recognized as one of the most significant biodiversity hotspots [1]. This biome harbors an extraordinary variety of plant species, many of which are rich in bioactive compounds, particularly phenolic compounds with remarkable therapeutic potential. Phenolic compounds belong to a diverse group of chemical structures characterized by an aromatic ring attached to a hydroxyl group. These compounds are well documented for their potent antioxidant and anti-inflammatory activities, which have been extensively studied due to their significant therapeutic potential. They are widely distributed in the Kingdom Plantae, occurring in a variety of species found in both leaves and fruits, where they play critical roles in defense mechanisms against oxidative stress, pathogens, and environmental stressors [2,3].

Among the numerous endemic fruits of the Cerrado, *Spondias mombin* L., commonly known as taperebá, cajá, or cajá-mirim, stands out for its promising biological activities, including gastroprotective effects demonstrated in animal models [4,5] and cytotoxic effects on ovarian cancer cell lines [6].

While the pulp of *S. mombin* is widely utilized in the food industry, the peel is often discarded as waste despite its high concentration of bioactive phenolic compounds, which are well known for their antioxidant properties and potential health benefits [7,8]. Previous research conducted by this group chemically characterized the phenolic compounds found in the peels of *S. mombin* using UPLC-MS/MS. The results demonstrated a significant content of polyphenolic compounds in the peels, emphasizing the high content of ellagic acid and quercetin, alongside considerable antioxidant potential. Moreover, this study successfully identified and quantified certain compounds in the *Spondias* genus for the first time [7]. These secondary metabolites play essential ecological roles in plants, such as by protecting them against pathogens, mitigating UV radiation damage, and reducing oxidative stress [9,10]. However, the clinical application of phenolic compounds is often limited due to their poor stability in gastric environments, low intestinal absorption, and susceptibility to degradation during storage [11,12].

To address these challenges, advanced delivery systems such as nanoencapsulation have garnered increasing attention. Nanostructured lipid carriers (NLCs) are lipid-based nanoparticles composed of a mixture of solid and liquid lipids at room temperature, such as oils and butters. Their structure, characterized by a solid matrix interspersed with more fluid regions, enhances overall structural flexibility and provides a versatile platform for the controlled release of bioactive compounds, with particles typically smaller than 100 nm, offering protection against environmental factors such as light and temperature [13,14,15]. Moreover, the surface properties of NLCs can be precisely tailored to support diverse therapeutic applications, ensuring stability and biocompatibility [16].

Simultaneously, the demand for sustainable and biocompatible encapsulation systems has driven the exploration of Natural Deep Eutectic Solvents (NaDESs). NaDESs represent a class of green solvents resulting from the combination of natural organic compounds, such as acids, sugars, amino acids, and choline derivatives. When mixed in specific proportions, these compounds interact through hydrogen bonding, forming a eutectic mixture with a melting point lower than that of the individual components. Due to this characteristic, NaDESs exhibit unique solubilization properties, enabling the dissolution of a wide range of bioactive molecules [17,18,19]. These solvents are not only non-toxic but also possess cryoprotective capabilities, stabilizing sensitive compounds during processes such as lyophilization, thereby enhancing their stability and storage potential [18,19]. Recent studies have highlighted the synergistic effects of integrating NaDESs with NLC formulations, resulting in improved stability and industrial scalability of nanoparticle systems [20,21].

Despite the rich antioxidant profile of *Spondias mombin* peel, its poor water solubility poses a significant challenge for formulation and bioavailability. In this context, its encapsulation within an NLC system using NaDESs as a solubilization medium for phenolic compounds extracted from *S. mombin* peel represents an innovative strategy to valorize this often-overlooked byproduct. NaDESs not only enhance the dissolution and stabilization of phenolic compounds but also contribute to the development of a more sustainable and biocompatible encapsulation system. This work sets a precedent for the development of scalable and environmentally friendly nanoformulations with potential applications in treating oxidative stress-related diseases, such as certain cancers, thereby expanding the therapeutic use of bioactives from Brazilian flora in global health applications [20].

The developed NLC-NaDES platform offers a versatile solution with potential applications in the pharmaceutical, nutraceutical, and cosmetic industries, driven by its capacity to stabilize and enhance the bioavailability of bioactive compounds. The synergy between nanostructured lipid carriers and NaDESs ensures efficient encapsulation and controlled release of phenolic compounds, making it particularly valuable for the treatment of oxidative stress-related pathologies, including neurodegenerative diseases, cancer, and chronic inflammatory disorders. The potent antioxidant properties demonstrated by the NLC-TAP-NaDES formulation underscore its suitability for therapeutic interventions aimed at mitigating oxidative cellular damage.

Furthermore, the system’s biocompatibility and cryoprotective capabilities expand its potential for cosmetic applications, such as anti-aging products and skin protectants, where oxidative stress plays a central role in cellular aging. In the nutraceutical field, this platform offers a promising vehicle for the delivery of natural antioxidants, thereby enhancing the functional value of dietary supplements. Additionally, the use of agricultural byproducts, such as *S. mombin* peels, aligns with the principles of a circular economy, promoting sustainability and resource efficiency. This green nanotechnology, therefore, bridges scientific innovation with environmental responsibility, opening new avenues for the valorization of underutilized natural resources while contributing to bioeconomy development.

## 2. Materials and Methods

### 2.1. Materials, Reagents and Samples

Analytical standard anhydrous sodium carbonate (≥99% purity) was obtained from Dinâmica Química Contemporânea Ltda., Indaiatuba, São Paulo, Brazil. Murumuru butter (*Astrocaryum murumuru*) and buriti oil (*Mauritia flexuosa*) were provided by Amazon Oil, Ananindeua, Pará, Brazil. Folin–Ciocalteu reagent, ethanol (≥99.5% purity), dimethyl sulfoxide (DMSO, ≥99.9% purity), Roswell Park Memorial Institute medium (RPMI-1640), Dulbecco’s Modified Eagle Medium (DMEM), 2,2-diphenyl-1-picrylhydrazyl (DPPH), 2,2′-Azino-bis(3-ethylbenzothiazoline-6-sulfonic acid) diammonium salt (ABTS), glycerol analytical standard, and Brij^®^ O10 were purchased from Sigma-Aldrich, St. Louis, MO, USA. Analytical standard choline chloride (≥98% purity) and anhydrous citric acid (≥99.5% purity) were obtained from Êxodo Científica, Sumaré, São Paulo, Brazil. Fetal bovine serum (FBS), penicillin-streptomycin solution (100 U/mL penicillin and 100 µg/mL streptomycin), and trypsin-EDTA (0.25%) were purchased from Gibco (Thermo Fisher Scientific), Waltham, MA, USA. The MTT reagent [3-(4,5-dimethylthiazol-2-yl)-2,5-diphenyl tetrazolium bromide] was obtained from Invitrogen (Thermo Fisher Scientific), Waltham, MA, USA. Phosphate-buffered saline (PBS, pH 7.4) was supplied by LaborClin, Pinhais, Paraná, Brazil.

### 2.2. Obtaining the Taperabá Fruits and Extract

The fruits of taperebá (*Spondias mombin* L.) were collected when they were ripe by the Brazilian Agricultural Research Corporation (Embrapa Cerrados (CPAC), located in Planaltina, Federal District—DF (15°54′16.50″ South and 47°22′37.49″ (West, at 855 m altitude). The characteristics of the area fall within the Cerrado biome. This project is registered in the National Genetic Heritage and Associated Traditional Knowledge Management System (SisGen) under numbers A002D4B and A235193. The fruits were peeled in a manual process, and the peels dried naturally at room temperature, protected from light, for 24 h. The extract was prepared according to Brito et al. (2022) [7].

### 2.3. Quantification of Phenolic Compounds and the In Vitro Antioxidant Activity of the Extract

The quantification of phenolic compounds in the extract was carried out through spectrophotometric analysis, being evaluated using the Folin–Ciocalteau method [22]. Calculations were made according to the gallic acid standard curve (y = 0.0012x + 0.0273, R^2^ = 0.9979) and the results were expressed as gallic acid equivalent (GAE) per L of taperebá peel extract.

Previous studies by this group have analyzed the phenolic compounds profile in these peels of *S. mombin* using ultra-performance liquid chromatography coupled with tandem mass spectrometry (UPLC-MS/MS), data previously described by De Brito et al. [7]. Chromatographic separation was carried out on a Waters Acquity UPLC system (Milford, MA, USA), and mass spectrometry detection was performed using a Waters Xevo TQMS instrument (Milford, MA, USA) equipped with an electrospray ionization (ESI) source using the method previously described by Vrhovsek et al. (2012) [22]. Multiple reaction monitoring (MRM) conditions were optimized using flow injections of individual metabolites; for most compounds, the Waters Intellistart software (version 4.1) performed automatic optimization, while for certain analytes, optimal cone voltages and collision energies were determined manually through collision-induced dissociation (CID) experiments

The in vitro antioxidant activity of the extract was evaluated through DPPH [23] and ABTS [24] free radical capture analysis. The results were expressed as Trolox equivalents (mM TEAC/100 mL extract of taperebá peel) and calculated from a standard curve.

### 2.4. Preparation of NaDESs

The natural analogs of deep eutectic solvents (NaDESs) were made with choline chloride, glycerol and citric acid (1:4:1), with 25% water. The mixture was heated to 80 °C until the reagents were completely dissolved [25]. After concentrating the ethanolic extract in a rotary evaporator, the concentrated extract was dissolved using the prepared NaDESs.

### 2.5. Development and Optimization of Formulations

To develop a formulation capable of nanoencapsulating the phenolic compounds extracted from taperebá peel, different proportions of surfactants and lipids were tested, following the methodology described by Silva [26], with specific adaptations for this study. Both systems with and without NaDESs were evaluated for their colloidal properties, thermal stability, and encapsulation efficiency.

### 2.6. Preparation of Lipidic and Aqueous Phases

Initially, formulations were prepared to standardize the individual components. For the lipidic phase, murumuru butter was used as the solid lipid, while buriti oil was included as the liquid lipid to enhance nanoparticle fluidity and stability. For the aqueous phase, the surfactant Brij^®^ O10 (Sigma-Aldrich, St. Louis, MI, USA) was dissolved in distilled water, with its concentration varied to minimize toxicity while optimizing colloidal stability.

### 2.7. Extract Solubilization

For non-NaDES formulations, the concentrated taperebá peel extract, obtained via rotary evaporation, was directly incorporated into the lipidic phase due to its poor water solubility. In contrast, for NaDES-based formulations, the extract was pre-diluted in a NaDES solution at a ratio of 1:6 (*w*/*v*), composed of choline chloride, citric acid, and glycerol. The resulting mixture was added to the lipidic phase after melting the murumuru butter, ensuring enhanced solubility and uniform dispersion.

### 2.8. Nanoformulation Production

The nanostructured lipid carriers (NLCs) were produced using a hot emulsification method. The lipidic and aqueous phases were heated separately to specific temperatures (70, 75, and 80 °C) to ensure homogeneity. After heating, the phases were combined and subjected to high-shear stirring to form an emulsion. Finally, the emulsion was rapidly cooled in an ice bath to solidify the lipid matrix and stabilize the nanoparticles.

### 2.9. Formulation Optimization

The formulations were evaluated based on their polydispersity index (PDI), measured by dynamic light scattering (Zetasizer, Malvern Panalytical), with acceptable values set at <0.25. Particle size was also measured to ensure dimensions below 200 nm, and macroscopic stability was assessed visually to check for phase separation or emulsion instability. Different proportions of the concentrated extract were tested to determine the optimal amount to incorporate, both in its pure form and when diluted in NaDESs. Additionally, the impact of varying NaDES volumes on the colloidal characteristics of the formulations was analyzed.

### 2.10. Assessment of Colloidal Properties

The Dynamic Light Scattering (DLS) technique was used to determine the mean particle hydrodynamic diameters and dispersion of the chosen NLCs formulations, using a ratio of 1:10 (*v*/*v*) in distilled water, and results expressed as an average of triplicates using the ZetaSizer (Nano ZS^®^, Malvern Instruments, Malvern, UK) at 25 °C.

The Zeta potential (mV) values of the selected NLCs were determined using the ZetaSizer equipment (Nano ZS^®^, Malvern Instruments, Malvern, UK) at 25 °C. The formulations were added to distilled water in a ratio of 1:10 (*v*/*v*).

### 2.11. Study of the Colloidal Stability of Formulations

**Figure 1:**
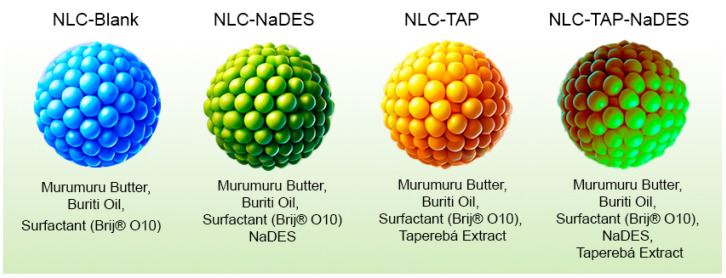
Composition of nanostructured lipid carrier (NLC) formulations. The table details the specific combinations of components used in each formulation, including murumuru butter (solid lipid), buriti oil (liquid lipid), surfactant Brij^®^ O10, natural deep eutectic solvents (NaDESs), and taperebá extract. Each formulation was designed to assess the individual and combined effects of these components on the physicochemical properties, stability, and bioactivity of the NLCs.

The formulations described in Figure 1 were subjected to a colloidal stability study, evaluating hydrogen-ion potential (pH) at 25 °C (pHmeter DM-22, Digimed, Sao Paulo, Brazil), as well as particle diameter, polydispersity, and zeta potential at 25 °C (Nano ZS^®^, Malvern Instruments, Malvern, UK), over time. These parameters were assessed on the day of formulation, after 24 h, and subsequently after 7, 15, and 30 days under different temperature conditions: room temperature (25 °C), high temperature (37 °C), refrigerated (−4 °C), and frozen (−20 °C).

### 2.12. Morphology of Nanoparticles (NPs)

The morphology of NLCs was evaluated by transmission electron microscopy (TEM) using the Transmission Electron Microscope JEM 1011 (JEOL, Tokyo, Japan), which was operated with an acceleration voltage of 80 kV. Suspensions were diluted 1:10 (*v*:*v*) with Milli-Q^®^ water and deposited directly onto carbon-coated grids used for observing the samples using an osmium tetroxide contrast. The microscope was operated in bright field mode with magnification above 10,000 times [27].

### 2.13. Assessment of Physicochemical Properties and Thermodynamic Stability of NLCs

The evaluation of physicochemical properties and thermodynamic stability of NCLs was carried out using thermogravimetric analysis (TGA). The NLCs that presented the most appropriate PDI and size results were analyzed. The contribution of NaDESs was also evaluated in NaDES samples without extract, and NLCs with murumuru butter, surfactants, and buriti oil (NLC-NaDES). In addition to NLC with extract dissolved in NaDES (NLC-TAP-NaDES) and NLC with extract without NaDESs (NLC-TAP), the pure lyophilized extract was also analyzed. The measurements were conducted using a differential thermal analysis (DTG-60, Shimadzu, Kyoto, Japan ). A heating rate of 10 °C/min was used, with a temperature range of 30–600 °C, under a nitrogen atmosphere with flow rate of 50 mL/min. The samples were previously lyophilized (L101 Liotop Lyophilizer, LABIOM São Carlos, Brazil) and analyzed in a platinum pan as a reference, using approximately 10 mg of material.

### 2.14. Study of the Stability of Total Polyphenols Contained in NLCs

The NLC formulations were subjected to study of the concentration stability of total polyphenols using spectrophotometric analysis, being evaluated using the Folin–Ciocalteau method [22,28] with adaptations. The nanostructures were subjected to agitation for 24 h in a 1% acetic acid solution, and then used for the Folin–Ciocalteau reaction. Before being evaluated in a spectrophotometer (U-3900H, Hitachi) at 750 nm, the reaction was centrifuged at 3500 rpm for 10 min.

The stability of the phenolic compounds was carried out on the day of formulation (day 0), and subsequently after 7, 15 and 30 days (days 7, 15 and 30). The samples were stored at different storage temperatures: ambient (25 °C), high (37 °C), refrigerated (−4 °C) and frozen (−20 °C).

### 2.15. Encapsulation Efficiency of Nanoparticles (NPs) 

After standardization, the NLCs were subjected to phenolic quantification. The effectiveness of the process was calculated as a percentage [28], according to Equation (1):

(1) $$\mathrm{EE}\%=\frac{\mathrm{Qobtained}}{\mathrm{Qtheoretical}}\times 100$$

EE% is the encapsulation efficiency of phenolic compounds in the nanoparticle, Qobtained is the amount of total polyphenols present after nanoencapsulation and Qtheoretical is the amount of total polyphenols that was added.

### 2.16. Fourier Transform Infrared Spectroscopy (FT-IR)

FT-IR spectra were acquired using a Vertex 70 spectrometer (Bruker Corporation, Billerica, MA, USA) in attenuated total reflectance (ATR) mode, with samples in liquid state. For analysis, 2 µL of each sample (without dilution) were deposited in a diamond crystal and the spectra were obtained in the region between 4.000 and 500 cm^−1^, with a resolution of 4 cm^−1^ and 64 scans, in absorbance mode. Data were acquired with OPUS 7.2 software (Bruker Corporation, Ettlingen, Germany) and processed with Origin Pro 2015 software (OriginLab Corporation, Northampton, MA, USA).

### 2.17. Cell Maintenance 

Fibroblasts (L929) were obtained from *Banco de Células do Rio de Janeiro* (BCRJ) and were cultured in RPMI medium, supplemented with 10% (*v*/*v*) fetal bovine serum (FBS) and 1% (*v*/*v*) antibiotic solution. The cells were maintained in an incubator under a humidified atmosphere with 5% CO_2_ at 37 °C throughout the entire experiment, ensuring stable culture conditions from cell seeding to the final analysis. Regular monitoring was performed to maintain cell viability and consistency during the experimental procedures.

### 2.18. Cultivation System and Obtaining of Zebrafish Embryos

The *Danio rerio* embryos utilized in the embryotoxicity test were supplied by the Zebrafish facility (Techniplast, Italy) of the Laboratory of Toxicologic Genetic of University of Brasília. The adults of zebrafish are maintained in an automated recirculating water system, stocked with filtered water by activated coal. The physical and chemical characteristics of the water are maintained in: pH 7.2–7.6; hardness 6.70 dH; temperature of 26 ± 1 °C; conductivity of 728 μS. The facility room disposes of a photoperiod of 12:12 h light/dark. The fishes are fed two to three times a day with commercial feed (SERAVipan^©^; Tetramin^©^) and live feed (nauplii de *Artemia salina*). The zebrafish embryotoxicity test does not require an animal ethic approval, because the embryogenesis up to 96 h is considered similar as an in vitro test, since the sensory system is not completely formed.

### 2.19. Viability of Fibroblasts (L929)

The 3-(4,5-dimethylthiazol-2-yl)-2,5-diphenyltetrazolium bromide (MTT) [29] assay was performed to analyze cell viability. The concentrations used were: 0.002 mg/mL; 0.008 mg/mL; 0.040 mg/mL; 0.200 mg/mL; 1.000 mg/mL; 5.002 mg/mL; and 25.008 mg/mL (mg NLC/mL).

Cell lines were plated in 96-well plates and treated for 24 h. In all groups, after the time points, the treatment was withdrawn and 150 µL of the MTT solution (0.5 mg/mL in culture medium) was added and incubated for 4 h at 37 °C. After incubation, this solution was removed and 200 µL of DMSO was added to dissolve the formazan crystals. The absorbance was measured at 540 nm using a microplate reader (SpectraMax^®^, Molecular Devices, San Jose, CA, USA) operated with the SoftMax Pro program (version 7.1, Molecular Devices).

### 2.20. Toxicity Test with Zebrafish Embryos (FET)

The embryo bioassays were based on the OECD protocol n. 236 [30] for toxicity assessment: Fish Embryo Toxicity—FET test. After collecting the embryos from the pawn breeding system, they were washed and immediately distributed into microplates with “test solutions” to ensure the beginning of exposure in the early stages. Exposure was carried out in 96-well microplates with 200 µL of each concentration. The tested concentrations were: 0 (control); 0.59 µg/mL; 1.18 µg/mL; 2.35 µg/mL; 4.70 µg/mL; 9.41 µg/mL; 18.81 µg/mL; and 37.63 µg/mL. The exposures were conducted in a climatic chamber with conditions identical to the culture room. The test solutions were prepared using zebrafish culture water (physical and chemical characteristics previously described). All bioassays were performed in triplicate with a total of 60 organisms per concentration and for 96 h.

### 2.21. Reactive Oxygen Species (ROS) Production Assay

L929 cells (1 × 10^3^ cells per well), both control and treated for 4 h with 10 µM of NLC-TAP-NaDES, were exposed to H_2_O_2_ to induce oxidative stress. After the treatments, the cells were gently washed with PBS (1×) and fixed with 3.5% formaldehyde for 15 min at room temperature. Subsequently, the cells were stained with 500 nM DAPI for nuclear labeling and with 5 µM CellROX^®^ Green Reagent (ThermoFisher^®^, Waltham, MA, USA) to detect intracellular reactive oxygen species (ROS).

Confocal images were captured using a Leica CS SP5 microscope, employing different z-stacks to acquire three-dimensional images of the cells. Wide-field images were obtained to analyze nuclear localization and the fluorescence of the CellROX^®^ Green Reagent (ThermoFisher^®^, Waltham, MA, USA) across the cell population. Quantitative analysis was performed on the fluorescence intensity of CellROX^®^ Green Reagent to evaluate whether NLC-TAP-NaDES treatment would reduce ROS production compared to control cells.

### 2.22. Statistical Analysis

Quantification of total phenolic, antioxidant activity and encapsulation efficiency of taperebá pell extract were calculated from a standard curve and the results were expressed as a mean of triplicates and standard deviation. ANOVA, Correlation Analysis and Tukey HSD Test were performed in the STATISTICA 10.0 program with a significance level of 5% (*p* < 0.05).

Therefore, the results of the FET were statistically analyzed using Sigma Plot 14.0. For normally distributed datasets, two-way ANOVA was used to detect intergroup differences. In cases of non-normal distributions, the Kolmogorov–Smirnov test for normality and Levene’s test for variance homogeneity were applied, followed by the Kruskal–Wallis test. Significant differences between tested concentrations and controls were determined using Dunnett’s or Dunn’s test for parametric or non-parametric data, respectively (*p* < 0.05).

The results of the formulation and stability of NLCs were expressed as a mean of triplicates and standard deviation. The MTT assay was performed in quadruplicate and in three independent experiments. The results obtained were analyzed using the Graphpad Prism 5.0 program and subjected to specific tests with statistical confidence of 95%. The Two Way Anova test was used.

## 3. Results and Discussion

### 3.1. Synthesis and Characterization

#### 3.1.1. Characterization of the Taperebá Pell Extract

Our work determined that the ethanolic extract of taperebá peels exhibits a remarkable total polyphenol content, corresponding to 2623 mg of gallic acid equivalents (EAG) per liter of fresh peel extract, which was subsequently utilized in the development of lipid nanostructures. The antioxidant capacity of this extract was rigorously evaluated using both DPPH and ABTS assays, yielding values of 258 mM TEAC and 495 mM TEAC per 100 mL of peel extract, respectively.

Complementary to these findings, previous investigations by our group employed UPLC-MS/MS to chemically characterize and quantify a diverse array of phenolic compounds in the peels of *Spondias mombin*. This comprehensive profiling revealed a substantial polyphenolic content, notably high in ellagic acid and quercetin, and marked the first successful identification and quantification of certain compounds within the *Spondias* genus [7]. In detail, the UPLC-MS/MS analysis enabled the quantification of several classes of phenolic compounds, including flavonols, phenylpropanoids, benzoic acid derivatives, coumarins, stilbenes, dihydrochalcones, flavones, and flavanones (Table 1).

Within the flavonol category, 14 distinct compounds were quantified, with quercetin being the most abundant, followed by myricetin and kaempferol-3-glucoside; noteworthy levels of syringetin and isorhamnetin-3-glucoside were also observed. The phenylpropanoid group was represented by cinnamic acid and its derivatives—encompassing various hydroxycinnamic acids and sinapyl alcohol—with chlorogenic acid present in the highest concentration, followed by p-coumaric and cinnamic acids. Among the benzoic acid derivatives, ellagic acid predominated over gallic acid in the taperebá peel extract. Additionally, the coumarin esculin was prominently detected, while among the stilbenes, cis-piceid was quantified at notably high levels, accompanied by significant amounts of trans-piceid and trans-resveratrol. The flavone fraction was characterized by the predominance of sinensetin and luteolin-7-O-glucoside, and the analysis further identified glycosylated hydroquinone (arbutin) [7].

In the quantitative analysis presented in Table 1, the phenolic composition of the extract reveals a pronounced heterogeneity across several compound classes. Among the benzoic acid derivatives, ellagic acid exhibits an exceptionally high concentration, followed by gallic acid and cinnamic acid, underscoring their potential pivotal role in mediating the extract’s antioxidant properties. Within the coumarin group, esculin is notably predominant. The phenylpropanoid fraction is characterized by a high level of chlorogenic acid, with p-coumaric acid and sinapyl alcohol further enhancing the spectrum of bioactive compounds. Notably, the stilbene category is distinguished by a substantial amount of cis-piceid, which contrasts with the lower concentrations of trans-piceid and trans-resveratrol, potentially indicating isomer-specific functional differences. The flavonoid spectrum is further diversified, with flavones like sinensetin and luteolin-7-O-glucoside and the flavanone naringenin playing notable roles. The flavonol group is particularly prominent, with quercetin and myricetin dominating the profile, complemented by significant levels of kaempferol-3-O-glucoside and isorhamnetin-3-O-glucoside, while additional compounds such as rutin and syringetin further contribute to the complexity. Moreover, the detection of arbutin as a hydroquinone derivative introduces an additional bioactive moiety to the extract. Collectively, these data illustrate a rich and diverse phenolic profile that is likely to underpin the extract’s multifaceted biological activities, thereby supporting its potential application in advanced therapeutic and cosmetic formulations [7].

This integrated characterization not only underscores the rich and diverse polyphenolic profile of taperebá peels but also highlights the extract’s potent antioxidant capacity and its potential applicability in the design of bioactive nanostructured lipid systems. Such findings pave the way for further exploration into the utilization of natural phenolic compounds in advanced material and pharmaceutical applications [7].

#### 3.1.2. Design of NLC’s 

Prior to the formulation of NLCs, the extract was concentrated using a rotary evaporator to reduce its volume and increase the concentration of bioactive compounds, thereby facilitating its subsequent incorporation into the nanoparticles. After concentration, the extract exhibited physical characteristics that made direct dissolution in water or conventional solvents challenging. To address this limitation, the concentrated extract was diluted in a pre-heated NaDES solvent, enabling more efficient incorporation into the lipid phase. As reported in the literature, NaDESs are known for their ability to solubilize phenolic compounds from natural extracts [20].

**Figure 2:**
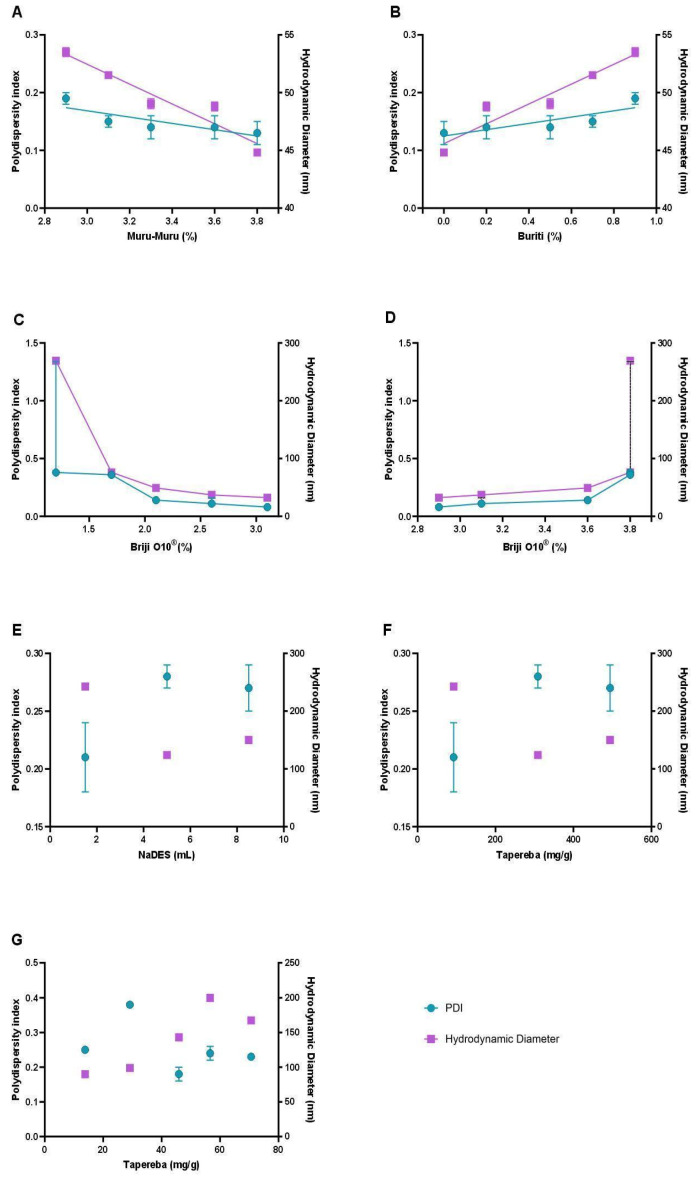
Influence of formulation parameters on the colloidal properties of nanostructured lipid carriers (NLCs). The graphs illustrate the effect of key variables, including murumuru butter percentage (**A**), buriti oil percentage (**B**), surfactant (Brij^®^ O10) concentration in different ranges (**C**,**D**), NaDES volume (**E**), and *Spondias mombin* extract concentration in NaDESs (**F**,**G**) on the polydispersity index (PDI) and hydrodynamic diameter (HD).

The NLC formulations were comprehensively evaluated using macroscopic analysis and detailed colloidal characterization, focusing on the influence of key variables such as lipid composition, surfactant concentration, extract content, and the incorporation of NaDESs containing the extract (Figure 2). Colloidal properties analyzed included the polydispersity index (PDI) and particle size, measured as the hydrodynamic diameter (HD). The PDI, ranging from 0 to 1, serves as an indicator of the uniformity in nanoparticle size distribution, with values closer to 0 signifying a more homogeneous system [31]. Particle size was precisely determined in nanometers (nm) using hydrodynamic measurements, providing a reliable metric for assessing the stability and scalability of the formulations.

Nanostructured lipid carriers (NLCs) represent the second generation of lipid nanoparticles and are systems for encapsulating drugs and active compounds, with a core structure formed by the combination of solid and liquid lipids at room temperature, stabilized by a surfactant layer. This characteristic provides the NLCs with greater fluidity in the lipid matrix, resulting in enhanced solubilization and stabilization of active compounds, as well as enabling their controlled release [32].

In this context, the formulation process for NLCs involves a careful selection of solid and liquid lipids to ensure the creation of small-sized, homogeneous particles. In this context, murumuru butter was selected as the solid lipid due to its high thermal stability and capacity to form crystalline lipid matrices, which are crucial for maintaining the structural integrity of the nanoparticles during storage and application [33,34].

The inclusion of buriti oil as the liquid lipid serves to enhance fluidity and improve the stability of the nanoparticles. Buriti oil is rich in monounsaturated fatty acids and antioxidants, such as tocopherols, which contribute to its stability and nutritional value [35,36]. The optimal ratio of murumuru butter to buriti oil is determined through systematic experiments that assess various parameters, including the polydispersity index (PDI), hydrodynamic diameter, and visual stability of the emulsions. Studies have shown that combinations with lower proportions of buriti oil and higher amounts of murumuru butter yield smaller and more stable particles, as indicated by PDI values remaining below 0.25, which is a benchmark for good emulsion stability.

The stability of emulsions is significantly influenced by the viscosity associated with the concentration of the dispersed phase, where higher viscosity can lead to improved stability [37]. Furthermore, the fatty acid profile of buriti oil, which includes a high content of oleic acid, contributes to its effectiveness in stabilizing emulsions and enhancing the overall performance of the NLCs [38,39]. The presence of antioxidants in buriti oil not only aids in the stability of the nanoparticles but also provides additional health benefits, making it a suitable candidate for various applications, including drug delivery systems [40]. These findings from systematic experiments underscore the importance of lipid selection and formulation parameters in achieving desirable characteristics in NLCs.

The integration of surfactants, such as Brij^®^ O10 (Sigma-Aldrich, St. Louis, MI, USA), was also critical for stabilizing the NLCs. This nonionic surfactant was selected for its compatibility with biological systems and low toxicity, essential features for health applications. Surfactant concentrations were adjusted to minimize toxicity without compromising colloidal stability. Analysis of the results revealed that lower surfactant levels increased particle size and PDI, indicating greater heterogeneity, whereas intermediate concentrations produced more uniform and stable particles.

A major innovation of this study was the incorporation of natural deep eutectic solvents (NaDESs) into the formulation of nanostructured lipid carriers (NLCs). Composed of glycerol, choline chloride, and citric acid, NaDESs were employed as a sustainable and biocompatible alternative to traditional solvents [41,42]. Beyond enhancing the solubility of phenolic compounds extracted from *Spondias mombin* peel, NaDESs contributed significantly to nanoparticle stabilization. This dual role was evidenced through improved colloidal stability and validated by infrared spectroscopy (FTIR), highlighting robust molecular interactions and protective effects that will be further detailed in subsequent sections [43,44].

Another pivotal aspect of the NLC development was the production method, which utilized hot emulsification followed by rapid cooling. This approach ensured the solidification of the lipid matrix, leading to the formation of nanoparticles with homogeneous sizes while effectively encapsulating phenolic compounds in a protective environment [45]. Key variables in the process, including extract concentration and NaDES percentage, significantly influenced the final properties of the NLCs [46]. Results demonstrated that higher emulsification temperatures (~80 °C) enhanced dispersion and reduced particle size in certain formulations. However, lower temperatures (~70 °C) were more appropriate for formulations containing NaDESs, accommodating the thermal sensitivity of these solvents and ensuring optimal nanoparticle stability [47,48].

The physicochemical characterization of the NLCs was another strength of the study. Zeta potential analyses demonstrated that the particles exhibited negative surface charges, promoting greater colloidal stability through electrostatic repulsion. Below, in Table 2, are the parameters of the final formulation with and without NaDESs (NLC-TAP-NaDES), which were defined as the main formulations used in this study. These data provide a comprehensive overview of the physicochemical and structural characteristics that underpin the functional properties of each formulation, enabling detailed comparisons and highlighting the effects of NaDESs’ presence on the systems analyzed.

#### 3.1.3. Nanoparticles (NPs) Morphology

**Figure 3:**
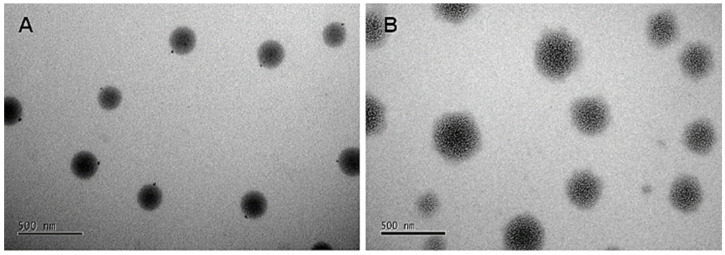
Photomicrograph obtained by transmission electron microscopy (TEM) for the taperebá pell extract encapsulated in NLCs. (**A**) NLC without NaDESs (NLC-TAP) and (**B**) NLC with NaDESs (NLC-TAP-NaDES).

The microscopic images in Figure 3 illustrate the morphology of NLCs without NaDESs (Figure 3A) and with NaDESs (Figure 3B). Both formulations exhibit a spherical shape, but Figure 3A displays a more organized structure, while Figure 3B reveals a diffuse morphology, likely influenced by NaDESs. This suggests that NaDESs interfere with the formation of lipid crystals, leading to structural disorganization.

These morphological differences provide critical insights into the impact of NaDESs on particle structure. Further evidence supporting this phenomenon, such as FTIR analyses showing reduced C-H band intensities in NaDES-containing formulations, will be discussed in detail later in this study.

#### 3.1.4. Colloidal Stability

**Figure 4:**
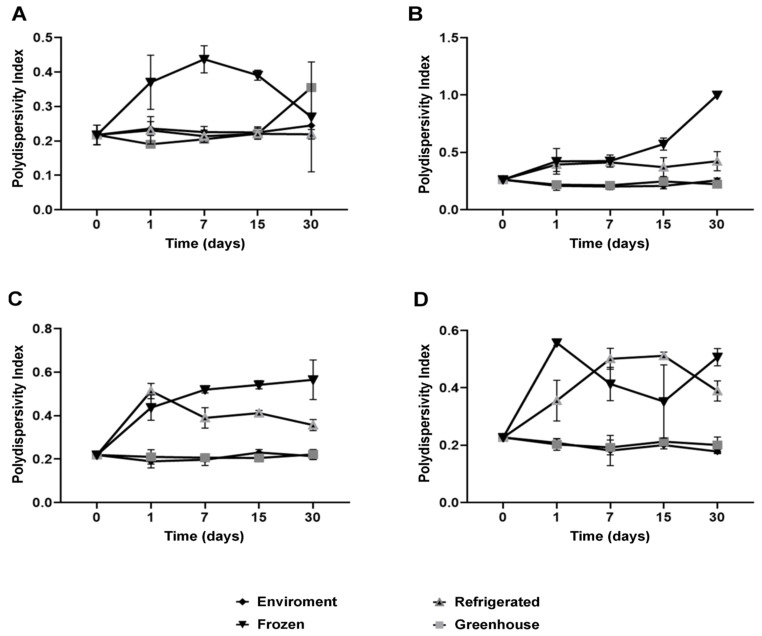
Polydispersity index (PDI) for each formulation stored at different temperature conditions over 30 days: NLC-Blank (**A**), NLC-NaDES (**B**), NLC-TAP-NaDESn (**C**), and NLC-TAP (**D**). Results are expressed as means of triplicates.

Colloidal stability is a critical parameter for the performance of nanostructured systems, and the results of this study emphasize the pivotal role of NaDESs in this context [32]. The polydispersity index (PDI), a key indicator of the homogeneity in particle size distribution, remained below 0.25 for most formulations under ambient and refrigerated conditions for up to 30 days (Figure 4). This indicates that the nanoparticles maintained high uniformity, essential for physical and functional stability.

Colloidal stability assays over time showed that NaDES-containing formulations maintained consistent particle sizes and PDI values under ambient and refrigerated conditions for up to 30 days. Under thermal stress (~40 °C), NaDES formulations outperformed their non-NaDES counterparts, highlighting their efficacy in preserving nanoparticle integrity and bioactive retention.

In summary, the development of NLCs in this study demonstrates a high degree of innovation, from component selection to the application of advanced production and characterization methodologies. The combinations of lipids, surfactants, and NaDESs were optimized to maximize encapsulation efficiency, stability, and functionality of the nanoparticles. These advances pave the way for the application of NLCs in various fields, including nutraceuticals, pharmaceuticals, and cosmeceuticals, while representing a milestone in the sustainable utilization of agricultural byproducts in cutting-edge technologies.

**Figure 5:**
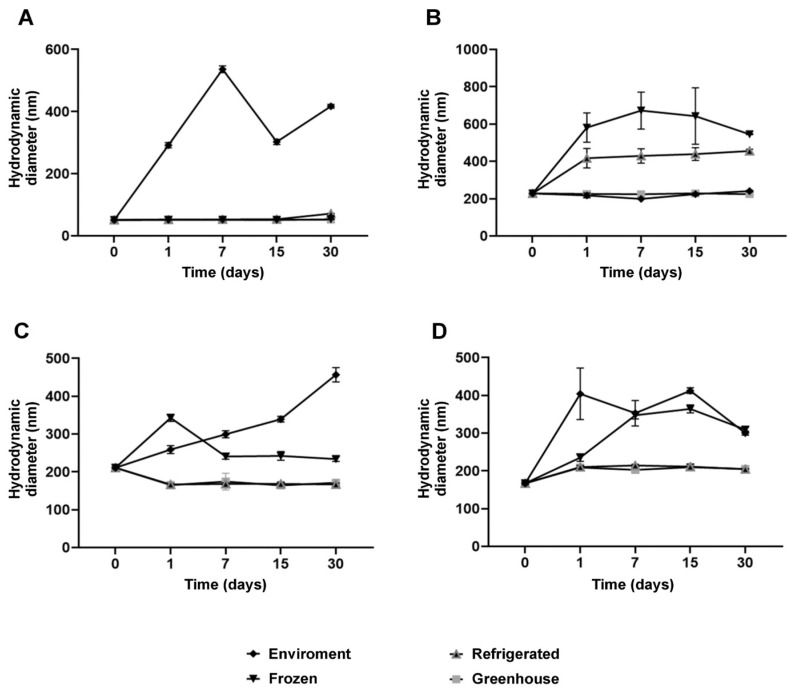
Hydrodynamic diameter (HD) size of the nanoparticle for each formulation stored at different temperature conditions over 30 days: NLC-Blank (**A**), NLC-NaDES (**B**), NLC-TAP-NaDES (**C**), and NLC-TAP (**D**). Results are expressed as means of triplicates.

Measurements of hydrodynamic diameter further reinforced the stabilizing effects of NaDESs. While formulations without NaDESs exhibited minimal size variations, NaDES-containing formulations demonstrated enhanced structural flexibility, observed through moderate size increases under thermal stress and freezing conditions (Figure 5). This behavior suggests that NaDESs modulate the lipid matrix, promoting greater thermal and functional stability.

#### 3.1.5. Differential Thermal Analysis (DTA) and Thermogravimetric Analysis (TGA)

**Figure 6:**
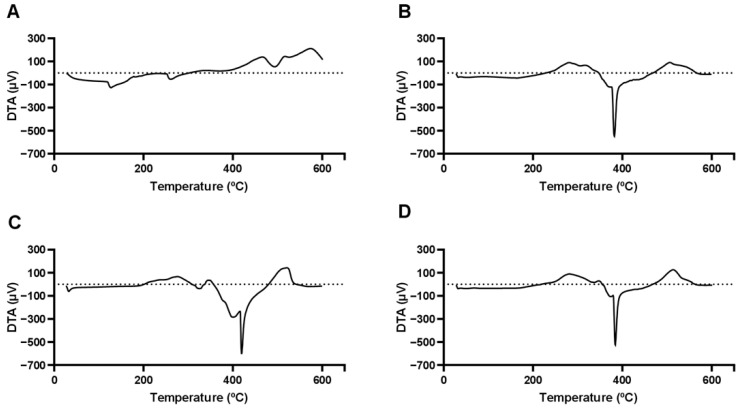
Differential thermal analysis (DTA) curves. (**A**) Tapereba extract; (**B**) NLC-NaDES; (**C**) NLC−TAP; (**D**) NLC−TAP−NaDES.

In the DTA curves shown in Figure 6A, it is possible to observe 3 endothermic events (127.27 °C, 260.70 °C, and 493.81 °C) and an exothermic event at 574.15 °C, which may be associated with the melting points of some components of the extract already quantified by this research group [7]. According to these quantification results, the taperebá peel extract contains several phenolic compounds, notably ellagic acid, quercetin, gallic acid and cis-Piceid [49].

**Figure 7:**
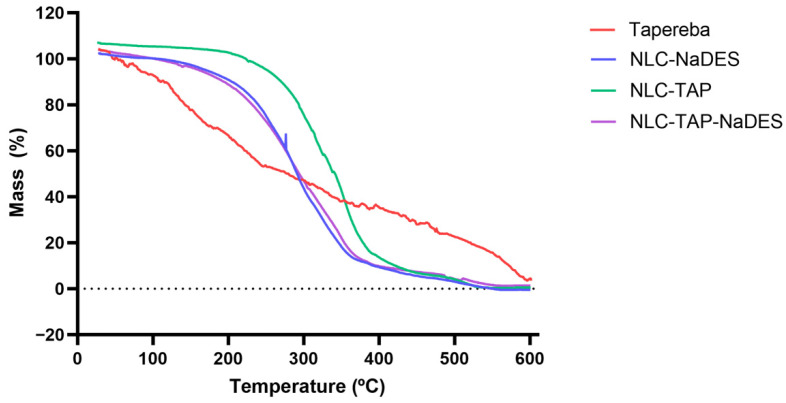
Thermogravimetric curves obtained for different materials and formulations: NLC-Blank, NLC−TAP, NLC−NaDES, and NLC−TAP−NaDES.

The other curves shown in Figure 6B,D represent an endothermic event around 380 °C, which shifts to higher values when NaDESs are not used. This result demonstrates that NaDESs enhance the dispersibility of the nanocarrier components, yielding a material with amorphous characteristics that are ideal for drug delivery systems [1]. This behavior is observed in the mass loss curves shown in Figure 7, indicating the influence of NaDESs on thermal stability.

The thermogravimetric analysis (TGA) graph (Figure 7) illustrates the mass loss of different formulations of nanostructured lipid carriers (NLCs) and the pure taperebá extract as a function of temperature. The *y*-axis represents mass loss (%), indicating thermal degradation, while the *x*-axis represents temperature (°C), ranging from 0 to 600 °C. The curves correspond to different samples: Taperebá extract (red), NLC-NaDES (blue), NLC-TAP (green), and NLC-TAP-NaDES (purple).

The pure taperebá extract (red) exhibits the earliest degradation, with mass loss beginning at approximately 100–150 °C and a significant decomposition phase extending up to 400 °C. This indicates the volatilization and thermal breakdown of bioactive phenolic compounds present in the extract.

The formulations containing NaDESs (NLC-NaDES in blue and NLC-TAP-NaDES in purple) display higher thermal stability, with delayed mass loss compared to the pure extract. Degradation occurs primarily between 200 and 400 °C, suggesting that NaDESs play a protective role in reducing the volatility of bioactive molecules and stabilizing the lipid matrix at elevated temperatures.

The NLC-TAP formulation (green), which lacks NaDESs, shows a slightly delayed decomposition compared to the pure extract, initiating at around 150–200 °C. However, its degradation occurs more rapidly than in NaDES-containing formulations, reinforcing the hypothesis that NaDESs contribute to enhanced thermal stability.

The investigation into the thermal stability of various formulations reveals significant insights regarding the efficacy of nanostructured lipid carriers (NLCs) compared to pure extracts. Specifically, pure extracts exhibit lower thermal stability, which can be attributed to their unprotected nature against thermal degradation. In contrast, NLC formulations demonstrate improved thermal resistance, which is critical for maintaining the integrity of bioactive compounds during processing and storage [50]. Among the NLC formulations, those incorporating natural deep eutectic solvents (NaDESs) show enhanced thermal protection. This stability is likely due to favorable molecular interactions between NaDESs and the lipid matrix, which effectively minimize premature volatilization of the active ingredients. Such interactions are crucial as they protect the bioactive compounds from thermal degradation and enhance their retention within the lipid matrix [51]. The presence of NaDESs can alter the physicochemical properties of the lipid carriers, leading to improved encapsulation efficiency and stability under thermal stress [52]. The findings underscore the potential applications of NLC-NaDES systems across various fields, including pharmaceuticals, cosmetics, and nutraceuticals. The improved stability and bioactive retention offered by these formulations make them suitable for thermal processing, which is often a requisite in the production of food and cosmetic products [53]. The ability of NLCs to withstand thermal degradation while preserving bioactive compounds positions them as a promising delivery system, ensuring that the therapeutic efficacy of the encapsulated agents is maintained even under challenging conditions [52,54]. In conclusion, the integration of NaDESs into NLC formulations not only enhances thermal stability but also opens avenues for their application in diverse industries, ensuring that bioactive compounds are effectively preserved and delivered.

#### 3.1.6. Retention and Stability of Phenolic Compounds

**Figure 8:**
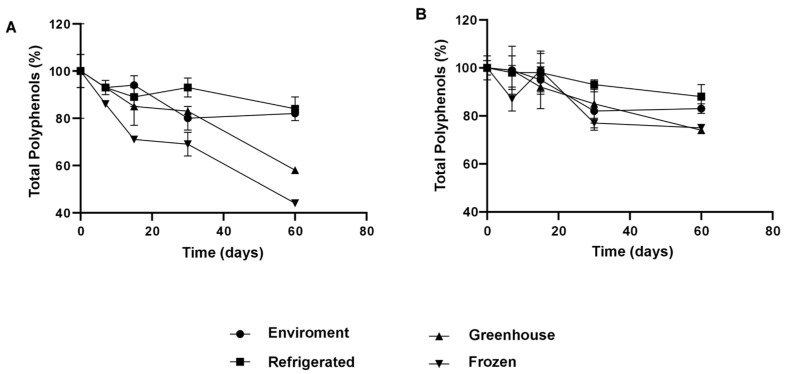
Representation of the concentration of total polyphenols (%) nanoencapsulated and stored at different temperature conditions. (**A**) NLC-TAP: formulation with murumuru butter, Brij^®^ O10, buriti oil and extract and (**B**) NLC-TAP-NaDES: formulation with murumuru butter, Brij^®^ O10, buriti oil and NaDES + extract.

The integration of NaDESs into NLCs resulted in significantly higher retention of phenolic compounds, even under adverse conditions (Figure 8). After 30 days, the NLC-TAP-NaDES formulation retained approximately 85% of its phenolic content under ambient conditions and 95% under refrigeration, whereas the NLC-TAP formulation showed inferior retention, particularly under thermal stress (~40 °C) and freezing. These results reflect the stabilizing role of NaDESs, which reduce the exposure of phenolic compounds to environmental factors and prevent oxidative degradation.

The incorporation of natural deep eutectic solvents (NaDESs) into nanostructured lipid carriers (NLCs) has been demonstrated to significantly enhance the retention of phenolic compounds, even when subjected to adverse environmental conditions (Figure 8). After 30 days, the NLC-TAP-NaDES formulation retained approximately 85% of its phenolic content under ambient conditions and 95% under refrigeration, whereas the NLC-TAP formulation showed inferior retention, particularly under thermal stress (~40 °C) and freezing. These results reflect the stabilizing role of NaDESs, which reduce the exposure of phenolic compounds to environmental factors and prevent oxidative degradation.

About the results of the percentage of total polyphenol content (TP) retention of nanoparticles formulated with taperebá peel extract, NLC-TAP (without the addition of NaDESs) and NLC-TAP-NaDES (with the addition of NaDESs), it was verified that, between the beginning of the test and the 15th day, there was no significant difference between the temperatures studied (*p* ≥ 0.05).

After 30 days of storage, it was found that refrigeration temperature showed the highest retention of TP in NLC-TAP (~95%), followed by ambient and stressful temperatures (~80%) (*p* ≥ 0.05). Ambient (~80%) and refrigeration (~85%) temperatures were those that obtained the highest values of TP retention in the nanoparticles at the end of the 60 days of testing (*p* ≥ 0.05).

NLCs without the addition of NaDESs (NLC-TAP) that were kept at freezing temperature were not able to protect the phenolic content (~40% TP retention) as effectively as under storage conditions at room temperature and refrigerated (~85%). This may be a consequence of the loss of colloidal stability of the nanoparticles due the phase separation during the freezing process. This separation leads to a high concentration of particles, which can generate irreversible aggregation and fusion of particulate material, in addition to the crystallization of ice, exerting mechanical stress on the system [41].

After 60 days of storage at stressful temperatures, NLCs without NaDESs (NLC-TAP) showed a significant decrease in TP retention (~50%). In theory, this type of entrapment of the active ingredient in nanostructures, which are made up of lipids in the solid and liquid phases (NLCs), has a lower melting point temperature than nanostructures made up only of lipids in the solid phase (SLN). This characteristic makes them unstable at stressful temperatures, but suitable for storage at room temperature [23]. Therefore, it is possible to suggest that there was instability of NLC-TAP when stored at ~40 °C, which promoted greater degradation of TP, as phenolic compounds are known to be thermolabile [6,37].

In general, at the end of this experiment, it was possible to verify that the NLCs with the addition of NaDESs obtained better results when compared to those without (*p* ≥ 0.05). The best storage conditions were refrigeration and room temperatures (~85% TP retention), followed by stressful and freezing temperatures (~75% TP retention), demonstrating interesting results on the use of NaDESs in these formulations.

Transmission electron microscopy (TEM) results (Figure 3) complement these findings, showing that NLC-TAP-NaDES particles exhibited a more diffuse morphology compared to the organized structure of NLC-TAP particles. This structural disorganization in NaDES-containing particles, also evidenced by the reduction in C-H band intensity in FTIR, enhances thermal flexibility and stability, particularly under extreme storage conditions.

Under freezing conditions, the cryoprotective effect of NaDESs was evident. While the NLC-TAP-NaDES formulation retained approximately 75% of its phenolic compounds after 30 days, the NLC-TAP formulation showed a reduced retention of about 50% (Figure 8). This behavior can be attributed to the ability of NaDESs to mitigate ice crystal formation and phase separation, phenomena known to promote aggregation and degradation of bioactives.

#### 3.1.7. FT-IR Spectroscopy

**Figure 9:**
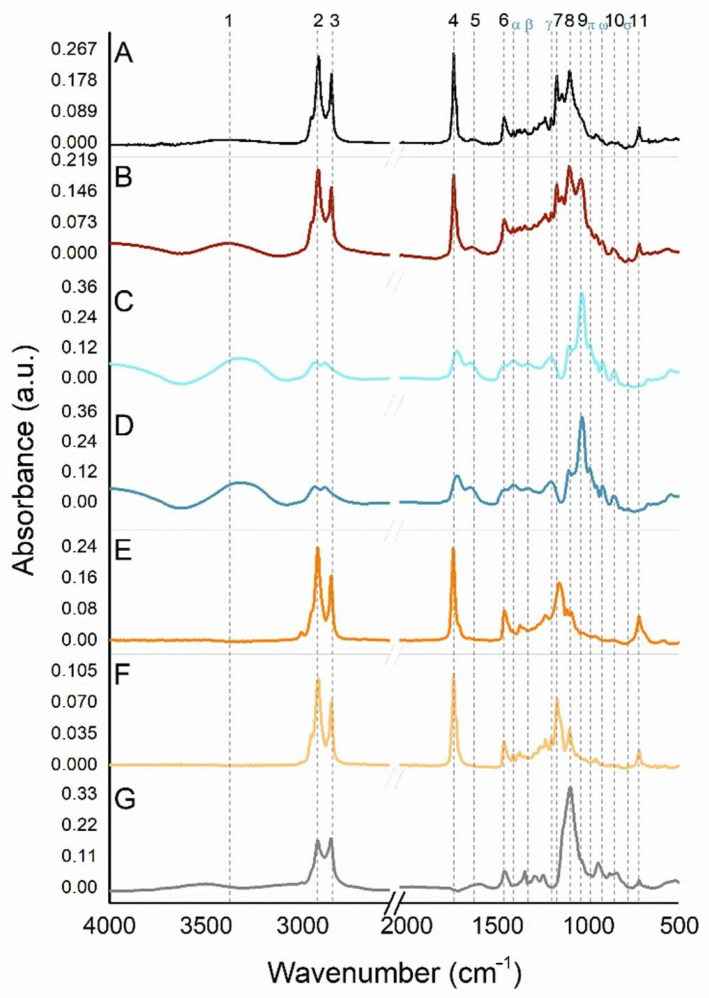
Fourier Transform Infrared (FT-IR) spectra. (**A**) NLC with extract and without NaDESs (NLC-TAP), (**B**) NLC with NaDESs and extract (NLC-TAP-NaDES), (**C**) NaDESs with extract, (**D**) NaDESs without extract, (**E**) Buriti oil, (**F**) Murumuru butter, and (**G**) Brij^®^ O10. The numbered dashed lines correspond to the peak’s positions, referring to sample B (nano extract with NaDESs). The dashed lines with Greek letters correspond to the peak’s positions referring to sample D (NaDESs).

Samples containing NaDESs with extract (Figure 9C) and NaDESs without extract (Figure 9D) showed minimal variations in relation to the identified peaks positions. The largest shift observed was ±5 cm^−1^. Thus, since the NLCs were formulated with *Spondias mombin* L. extract, the reference values considered to this analysis will be given from the NaDESs with extract sample (C). The signatures (sig.) found are in consonance with the literature and correspond to the presence of glycerol, citric acid, and choline chloride. The broad band at 3321 cm^−1^ (sig. 1) is related to ν(OH), the narrow peaks at 2934 (sig. 2) and 2890 cm^−1^ (sig. 3) assign to ν_as_(CH_2_) and ν_s_(CH_2_), respectively. 1721 (sig. 4) and 1646 cm^−1^ (sig. 5) can be assigned to ν(C=O). In the fingerprint region (1500–500 cm^−1^), the narrow and strong peak situated in 1035 cm^−1^ (sig. 9) refers to ν_as_(C-C-O) assignment, and can be associated with glycerol, as well as choline chloride presence. The additional medium and weak peaks in this region at 1411 (sig. α), 1331 (sig. β), 1205 (sig. γ), 1107 (sig. 8), 991 (sig. π), 921 (sig. ω), 860 (sig. 10), and 780 cm^−1^ (sig. σ) may be accredited, correspondingly, to δ(C-O-H), ρ(C-O-H), ν(C-C-O), δ(OH), ν(CN), δ(OH), ν_s_(C-C-O), and ρ(CH_2_). The taperebá extract did not indicate any influence regard to the molecular pattern in the IR spectra containing NaDESs.

As occurs with NaDES samples, several functional groups with the same signature are present in the buriti oil, murumuru butter, and some in Brij^®^ O10 IR spectra. What takes place with Brij^®^ O10 signatures is that this surfactant comes from vegetable-based fatty ethers. Brij^®^ O10 similar signatures to the lipid part used in the formulation (buriti and murumuru) correspond to ν_as_(CH_2_), ν_s_(CH_2_), δ(CH_2_), ν(C-C-O), and ρ(CH_2_), respectively, 2920 (sig. 2), 2853 (sig. 3), 1463 (sig. 6), 1101 (sig. 8), and 723 cm^−1^ (sig. 11). The narrow medium peaks (absent in Brij^®^ O10) around 1741 and 1744 cm^−1^ (sig. 4), and 1172 and 1161 cm^−1^ (sig. 7), for murumuru butter and buriti oil, correspond, subsequently, to ν(C=O) and ν(C=C-C-O).

Comparing the obtained NLCs IR spectra, it is observed, at ν(OH) (3367 and 3375 cm^−1^, for NLC-TAP (Figure 9A) and NLC-TAP-NaDES (Figure 9B), respectively), the difference on the band shape between the samples. For NLC-TAP-NaDES this broad band (sig. 1) becomes defined, as well as presenting greater relative intensity, indicating that the amount/concentration of these (OH) groups is also higher. This contribution can be given due to the substantial availability of hydroxyl compounds related to glycerol, citric acid, and choline chloride, since water content used in the formulation is also smaller than NLC-TAP. The peaks corresponding to ν_as_(CH_2_) and ν_s_(CH_2_) (2914 and 2927 cm^−1^ (sig. 2), and 2855 and 2853 cm^−1^(sig. 3)), for NLC-TAP-NaDES and NLC-TAP, resemble the same peaks present in the precursors (butter, oil and tensioactive). The shifts that occur in the spectral region between 4000 and 2500 cm^−1^ (vibrational modes ν(OH), ν(CH) or ν(NH)) may happen due to the overlapping of organic molecules involved in the reaction, as is the case of shifts (±10 cm^−1^) situated in ν(OH) and ν_as_(CH_2_), when comparing the NLCs. The other signatures found (ν(C=O) (sig. 4), δ(CH_2_) (sig. 6), ν(C=C-C-O) (sig. 7), and ρ (CH_2_) (sig. 11)), in both samples, are in line with the buriti oil, and murumuru butter. The exclusive peaks found in the NLC-TAP-NaDES sample (1030 (sig. 9), 991 (sig. π), 923 (sig. ω), and 864 cm^−1^ (sig. 10), subsequently, ν_as_(C-C-O), δ(OH), ν(CN), and ν_s_(C-C-O), ascribed to NaDESs. Additionally, it should be evidenced that the presence of glycerol among the exclusive signatures mentioned for NLC-TAP-NaDES is relevant, since this compound, besides being coming from natural/vegetable bases, is related to several technological applications to the pharmaceutical (capsules, antibiotics, antiseptics), and cosmeceutical (moisturizers, deodorants, and makeup) industries.

### 3.2. Embryotoxicity, Cytotoxicity Testing and In Vitro ROS Production

**Figure 10:**
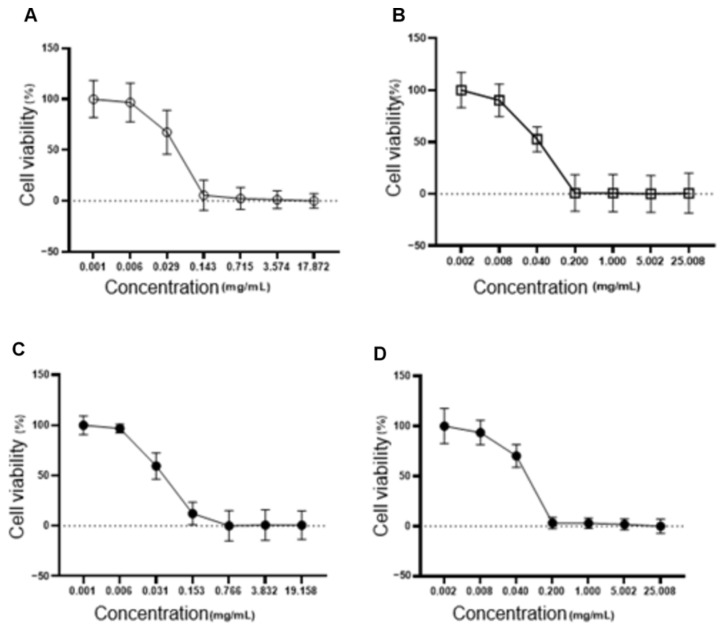
Cytotoxicity assays in fibroblasts (L929). The graphs illustrate the dose-dependent effect of different NLC formulations on cell viability (%), with IC50 values for each formulation. The IC50 values obtained were 39.95 µg/mL for NLC−Blank (**A**), 40.75 µg/mL for NLC−NaDES (**B**), 40.10 µg/mL for NLC−TAP (**C**), and 56.48 µg/mL for NLC−TAP−NaDES (**D**). The NLC−TAP−NaDES formulation exhibited significantly lower cytotoxicity compared to other formulations (*p* < 0.05), supporting the hypothesis that NaDESs reduce the direct interaction of phenolic compounds with cellular targets, thereby enhancing biocompatibility. These results highlight the potential of NaDES-containing NLCs in mitigating toxicity while maintaining phenolic stabilization.

Cytotoxicity assays in fibroblasts (L929) showed a higher IC50 for the NLC-TAP-NaDES formulation (56.48 µg/mL) compared to formulations without NaDESs (NLC–TAP), indicating reduced cytotoxicity in normal cells (Figure 10). These results align with the hypothesis that NaDESs enhance the encapsulation efficiency and stability of phenolic compounds, reducing their direct interaction with cellular targets and mitigating toxicity. The combined results of these bioassays suggest that while NaDESs-containing NLCs offer significant advantages in phenolic stabilization, further studies are needed to optimize their concentrations and evaluate their long-term effects in more complex biological systems. The cytotoxicity of NLCs was also tested on fibroblasts, and is represented in Figure 10. The IC50 values obtained were 39.95 µg/mL for NLC-Blank; 40.75 µg/mL for NLC-NaDES; 40.10 µg/mL for NLC-TAP; and 56.48 µg/mL for NLC-TAP-NaDES, the latter being the only one that showed a statistical difference (*p* < 0.05).

**Figure 11:**
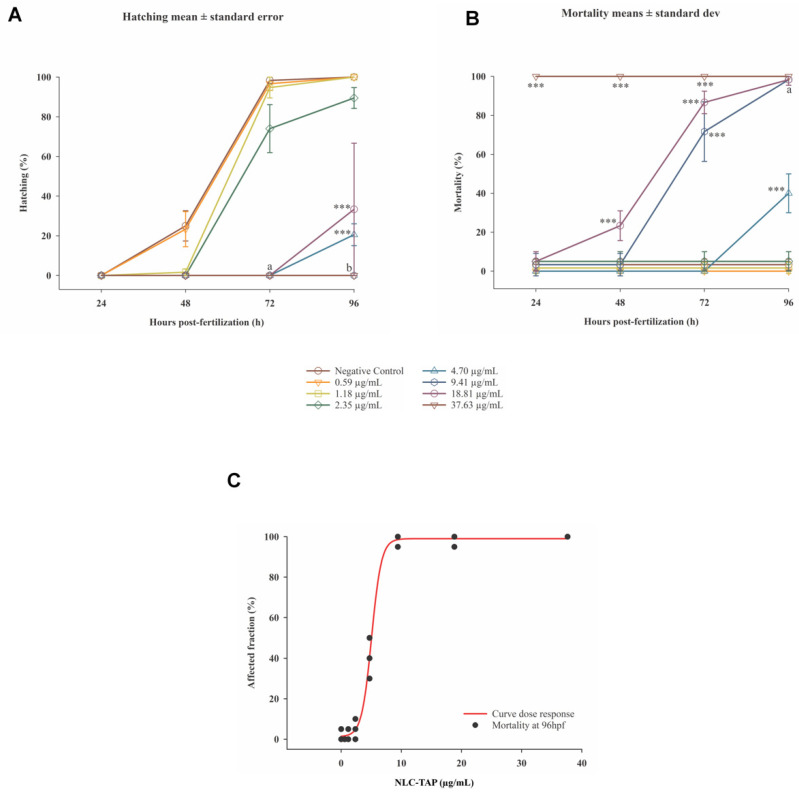
Graphics depicting the embryotoxicity test of NLC-TAP in zebrafish. (**A**). Chart illustrating the hatching rate over the 96 h exposure period. (**B**). Graph depicting the mortality rate throughout the 96 h exposure period. Significant differences compared to the negative control are indicated by *** *p* < 0.001, where both a and b also signify statistical differences of *p* < 0.001. In (**A**) concentrations of 4.70 μg/mL, 9.41 μg/mL, 18.81 μg/mL, and 37.63 μg/mL are associated, with data presented as mean ± standard error. In (**B**) concentrations of 9.41 μg/mL and 37.63 μg/mL are presented, with data shown as mean ± standard deviation. (**C**) Concentration-response curve (mortality) of organisms exposed to NLC-TAP for 96 h, revealing an LC50 of 5.05 μg/mL—Model: sigmoidal—4 parameters. R^2^ = 0.99.

The embryotoxicity and cytotoxicity assays revealed important awareness into the biocompatibility and potential therapeutic window of NLC-TAP-NaDES. Zebrafish embryo tests showed a dose-dependent toxicity profile, with an LC50 of 5.05 μg/mL for the NLC-TAP-NaDES formulation. The results indicated significant mortality at higher concentrations, particularly at 37.63 μg/mL, where 100% lethality was observed within 24 h (Figure 11).

**Figure 12:**
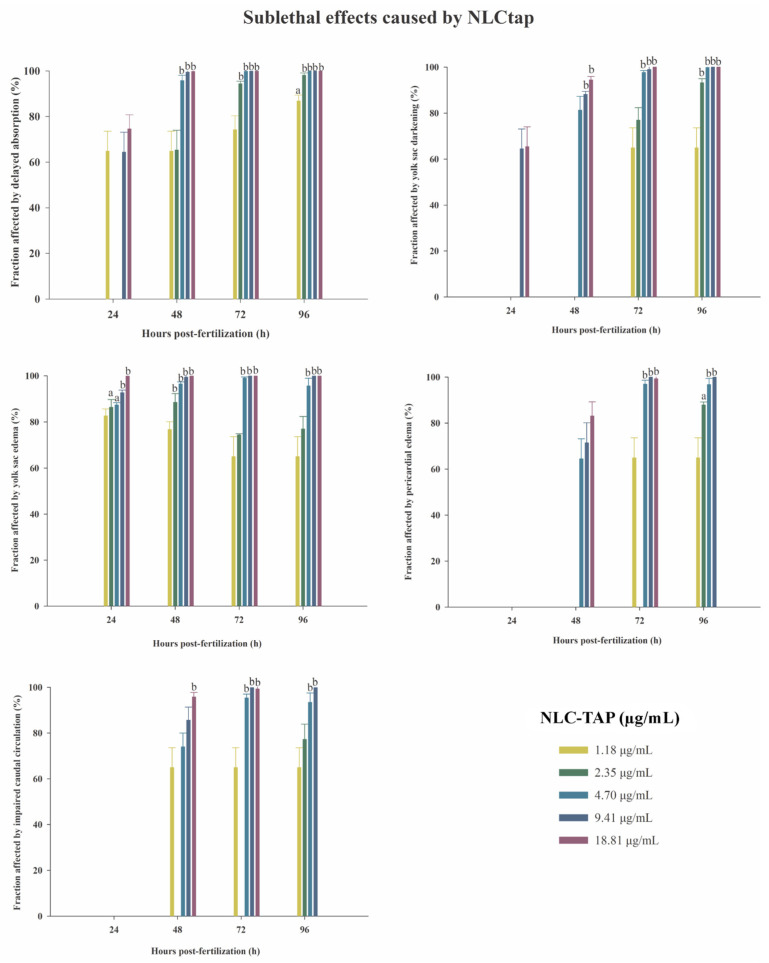
Graphics of sublethal effects caused by various concentrations of NLC-TAP during the embryo-toxicity test in zebrafish. Delayed absorption of the yolk sac, darkening of the yolk sac, edema in the yolk sac, edema in the pericardium, and alteration in caudal circulation. Significant differences are represented by letters (a and b), where a indicates *p* < 0.005 and b indicates *p* < 0.001. Data are presented as mean ± standard deviation.

**Figure 13:**
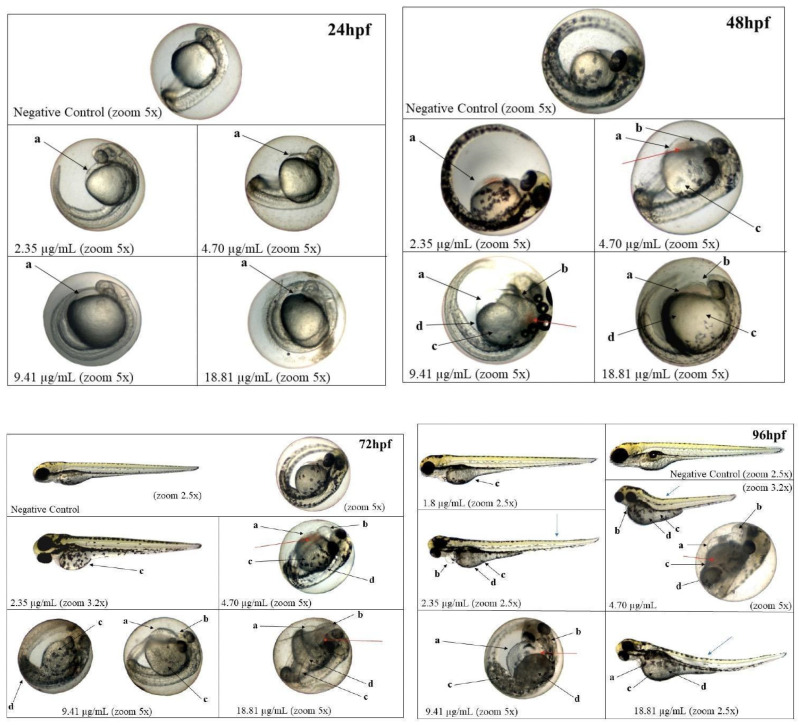
Photodocumentation of organisms exposed for 96 h to NLC-TAP. Black arrows—a: yolk sac edema; b: pericardial edema; c: delayed absorption; d: yolk sac darkening. Blue and red arrows indicate sublethal changes that did not show statistically significant differences. Blue arrows: alterations in the notochord. Red arrows: blood stasis.

These findings underscore the importance of optimizing formulation concentrations for safe application in biological systems. Sublethal effects, such as delayed yolk sac absorption, yolk sac darkening, and pericardial edema, were observed predominantly at higher concentrations (Figure 12). Notably, the NLC-TAP-NaDES formulation demonstrated a less pronounced impact on embryonic development compared to formulations without NaDESs, suggesting a potential mitigating role of NaDESs in modulating the bioavailability and toxicity of phenolic compounds. It is also possible to observe the photodocumentation of organisms exposed to NLC-TAP in Figure 13.

The results of the embryotoxicity and cytotoxicity assays conducted on nanostructured lipid carriers (NLCs) containing natural deep eutectic solvents (NaDESs) provide compelling evidence for the viability of these formulations as a platform for the delivery of bioactive compounds. The findings indicate a significant reduction in cytotoxicity when these NLCs are administered to normal cells, suggesting a favorable biocompatibility profile [55]. This characteristic is particularly important in the context of drug delivery systems, where the safety of the carrier is paramount to ensure that therapeutic agents can be delivered effectively without causing undue harm to healthy tissues.

Moreover, the moderated impact on embryonic development observed at sublethal doses further supports the notion that NaDES-containing NLCs can be designed to minimize adverse effects during critical stages of development [56]. This is a crucial consideration, especially in applications involving reproductive health and developmental therapeutics. The ability to maintain a balance between efficacy and safety is a hallmark of successful drug delivery systems, and the data presented in this study suggest that NaDES-containing NLCs may achieve this balance effectively.

However, it is essential to note the dose-dependent toxicity observed at higher concentrations of these formulations. This finding underscores the necessity for meticulous optimization of the NLC formulations to ensure that they can be safely utilized across a range of concentrations [57]. The implications of this observation are significant; it highlights the importance of conducting thorough dose–response studies to establish safe dosage thresholds that maximize therapeutic benefits while minimizing potential risks.

**Figure 14:**
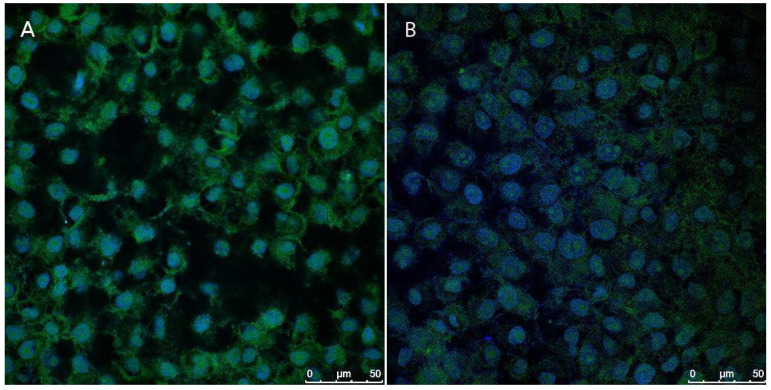
The fluorescence images of reactive oxygen species (ROS) production, assessed using the CellROX^®^ Green Reagent assay after treatment with NLC-TAP-NaDES for 24 h, are shown. Panel (**A**) displays the control cells, while panel (**B**) depicts the cells treated with NLC-TAP-NaDES. ROS production is indicated in green, while DAPI staining, marking the cell nuclei, is shown in blue.

Figure 14 presents fluorescence images depicting the production of reactive oxygen species (ROS) in cells initially subjected to H_2_O_2_-induced oxidative stress, followed by treatment with the NLC-TAP-NaDES formulation in the treated group. Panel A represents the control group, where stressed cells were left untreated, while panel B shows the cellular response to the NLC-TAP-NaDES treatment.

The results highlight a markedly lower fluorescence signal in panel B, demonstrating the formulation’s ability to mitigate the intracellular accumulation of ROS caused by H_2_O_2_. This antioxidant effect is attributed to the bioactive phenolic compounds derived from the peel extract of taperebá (*Spondias mombin*), which are rich in ellagic acid and quercetin—well-known radical scavengers. Previous studies by our group have shown that taperebá peel extract exhibits a high polyphenol content (2623 mg GAE/L) and strong antioxidant activity, as assessed by the DPPH (258 mM TEAC/100 mL) and ABTS (495 mM TEAC/100 mL) assays [7]. These properties make the extract a promising source for antioxidant applications.

Moreover, the role of NaDESs combined with the NLC system was pivotal in enhancing the stability and bioavailability of the bioactive compounds. The NLC-NaDES system effectively protected polyphenols from oxidative degradation and enabled controlled release of antioxidants, targeting their action to mitigate the oxidative damage induced by H_2_O_2_. Consequently, treated cells exhibited a significantly reduced oxidative load compared to the control, as evidenced by the diminished fluorescence intensity associated with ROS production.

This study underscores the capability of NLC-NaDES-based formulations to alleviate oxidative damage under conditions of pre-induced stress, as demonstrated here with H_2_O_2_. The approach leverages underutilized by-products, such as taperebá peels, aligning with the principles of sustainability and circular bioeconomy. The proposed technology represents a substantial advancement in the development of innovative antioxidant therapies, with potential applications in addressing oxidative stress-related diseases, including cancer, neurodegenerative disorders, and chronic inflammation.

These findings also support the feasibility of employing this platform in preclinical studies to evaluate its efficacy under more complex conditions and, eventually, its application in pharmaceutical and nutraceutical formulations aimed at managing redox imbalance in living systems. The combination of bioactive natural extracts, NaDESs, and NLC exemplifies a modern paradigm of green nanotechnology with broad applications and global impact in the field of antioxidant science.

To further substantiate the therapeutic potential and safety of NaDES-containing NLCs, future research should prioritize in vivo assessments. These studies are critical for understanding the pharmacokinetics, biodistribution, and long-term effects of these formulations within a living organism [58]. In vivo models can provide insights that are not attainable through in vitro studies alone, particularly regarding the interactions between the NLCs and biological systems, including immune responses and metabolic pathways.

Additionally, long-term evaluations are necessary to assess the chronic effects of these formulations, particularly in relation to their cumulative exposure and potential for toxicity over extended periods [59]. Such studies will be instrumental in determining the feasibility of these NLCs for clinical applications, especially in health-related fields where sustained delivery of bioactive compounds is desired.

In conclusion, while the preliminary results are promising, the path forward requires a comprehensive approach that includes optimization of formulation parameters, rigorous in vivo testing, and long-term safety evaluations. By addressing these critical areas, researchers can pave the way for the successful application of NaDES-containing NLCs in various therapeutic contexts, ultimately contributing to advancements in drug delivery systems and improving patient outcomes.

### 3.3. Sustainable Implications and Future Applications

The integration of nanostructured lipid carriers (NLCs) with natural deep eutectic solvents (NaDESs) represents a significant advancement in the utilization of agricultural byproducts, particularly the peel of *Spondias mombin*. This innovative approach aligns with global sustainability trends and circular economy practices, emphasizing the efficient use of natural resources [60,61]. Agricultural and food industries produce substantial volumes of underutilized byproducts, which are often discarded as waste. The transformation of *S. mombin* peel, rich in bioactive phenolic compounds, into a valuable resource through advanced nanotechnology exemplifies the potential of co-product-based innovations to address critical environmental challenges, such as waste accumulation and resource inefficiency [62].

The application of NLCs and NaDESs not only enhances the bioavailability and stability of phenolic compounds but also reduces the environmental footprint associated with traditional manufacturing processes. By leveraging agricultural byproducts and eliminating the need for toxic solvents, this approach provides a sustainable model for industrial innovation [63]. This synergy between green solvents and nanostructured delivery systems illustrates how environmentally friendly and biocompatible technologies can support the production of scalable solutions that align with green chemistry principles [64,65].

From a neo-industrialization perspective, the valorization of *S. mombin* peel and similar co-products contributes to building resilient value chains that prioritize resource efficiency, reduced carbon emissions, and technological innovation. These efforts resonate with policies promoting green industrial development, particularly in emerging economies where agricultural byproducts represent an abundant yet untapped resource [66,67]. Furthermore, this study highlights the potential for such technologies to catalyze the development of next-generation materials that address global health challenges, integrating the principles of bioeconomy, circular economy, and technological innovation [68,69].

Future research should focus on scaling these technologies for industrial applications, exploring their potential in broader sectors such as environmental remediation, food preservation, and renewable energy systems. Collaborations between academia, industry, and policymakers will be essential to accelerate the adoption of co-product-based innovations, ensuring they contribute to global efforts towards a more sustainable and equitable industrial future [70,71].

## 4. Conclusions

The findings of this study demonstrate the successful development of nanostructured lipid carriers (NLCs) incorporating natural deep eutectic solvents (NaDESs) as a novel and sustainable platform for the encapsulation and stabilization of bioactive phenolic compounds extracted from *Spondias mombin* peel. The careful optimization of lipid ratios, surfactant concentrations, and the integration of NaDESs not only improved the physicochemical properties of the nanoparticles but also enhanced the retention and stability of the encapsulated bioactives under varying environmental conditions. The use of murumuru butter and buriti oil as lipid components, coupled with Brij^®^ O10 (Sigma-Aldrich, St. Louis, MI, USA) as a surfactant, resulted in formulations with low polydispersity indices, stable zeta potentials, and hydrodynamic sizes suitable for advanced therapeutic and industrial applications.

The inclusion of NaDESs, comprising glycerol, choline chloride, and citric acid, played a pivotal role in solubilizing phenolic compounds, facilitating their incorporation into the lipid phase, and stabilizing the nanoparticles through robust molecular interactions. These interactions, evidenced by FTIR and corroborated by TEM analysis, underscored the protective microenvironment created by NaDESs, which mitigated oxidative and thermal degradation while providing flexibility to the lipid matrix. Moreover, the cryoprotective properties of the NaDESs contributed to improved nanoparticle stability under freezing conditions, further broadening the potential applications of these formulations.

The methodological advances in the production of NLCs, utilizing hot emulsification and rapid cooling, ensured consistent particle sizes and encapsulation efficiency, while the thorough characterization of colloidal properties validated the robustness and scalability of the formulations. The integration of NaDESs into NLCs represents a paradigm shift in the valorization of agricultural byproducts, aligning with global efforts towards sustainability, circular economy practices, and green industrial innovation. By transforming *S. mombin* peel—a byproduct often discarded as waste—into a high-value ingredient for health-related applications, this study highlights the potential for co-product-based technologies to address critical environmental and industrial challenges.

Future research should explore the scalability of these formulations to industrial levels, investigate their efficacy in diverse biological systems, and extend their application to other bioactive compounds. Additionally, fostering interdisciplinary collaborations between academia, industry, and policymakers will be essential to realize the full potential of these technologies, ensuring they contribute to a more sustainable and equitable future. This work not only sets a foundation for advanced delivery systems but also exemplifies how nanotechnology, coupled with sustainable practices, can drive innovation across health and industrial sectors.

## Figures and Tables

**Table 1:** Quantification of metabolites from taperebá peel (µg/g) by UPLC-MS/MS Table adapted from De Brito et al. [7].

Category	Compound	Concentration (µg/g)
Benzoic acid derivatives	2,5-Dihydroxybenzoic acid	0.102 ± 0.090
Benzoic acid derivatives	Cinnamic acid	1.254 ± 0.040
Benzoic acid derivatives	Ellagic acid	79.080 ± 3.272
Benzoic acid derivatives	Gallic acid	21.994 ± 0.361
Benzoic acid derivatives	Methyl anthranilate	0.003 ± 0.002
Benzoic acid derivatives	Methyl gallate	0.031 ± 0.010
Benzoic acid derivatives	Syringaldehyde	0.136 ± 0.053
Benzoic acid derivatives	Vanillic acid	0.006 ± 0.003
Benzoic acid derivatives	Vanillin	0.869 ± 0.080
Coumarins	Daphnetin	0.377 ± 0.121
Coumarins	Esculin	4.016 ± 0.371
Coumarins	Fraxin	0.088 ± 0.030
Coumarins	Scopoletin	0.382 ± 0.161
Phenylpropanoids	Caffeic acid	0.239 ± 0.031
Phenylpropanoids	Chlorogenic acid	10.236 ± 0.211
Phenylpropanoids	Cryptochlorogenic acid	0.141 ± 0.012
Phenylpropanoids	Ferulic acid	0.163 ± 0.025
Phenylpropanoids	Neochlorogenic acid	0.129 ± 0.011
Phenylpropanoids	p-Coumaric acid	1.868 ± 0.211
Phenylpropanoids	Sinapyl alcohol	1.142 ± 0.042
Phenylpropanoids	trans-Coutaric acid	0.036 ± 0.031
Stilbenes	cis-Piceid	21.106 ± 1.330
Stilbenes	trans-Piceid	3.577 ± 0.181
Stilbenes	trans-Resveratrol	0.650 ± 0.022
Dihydrochalcones	Phloretin	0.017 ± 0.004
Dihydrochalcones	Phloridzin	0.215 ± 0.041
Dihydrochalcones	Trilobatin	0.051 ± 0.011
Flavones	Apigenin	0.035 ± 0.011
Flavones	Hesperidin	0.129 ± 0.071
Flavones	Luteolin	0.214 ± 0.011
Flavones	Luteolin-7-O-Glucoside	1.185 ± 0.081
Flavones	Sinensetin	1.453 ± 0.031
Flavanones	Naringenin	1.037 ± 0.061
Flavonols	Isorhamnetin	0.769 ± 0.051
Flavonols	Isorhamnetin-3-O-glucoside	1.788 ± 0.081
Flavonols	Kaempferol	0.374 ± 0.061
Flavonols	Kaempferol-3-O-glucoside	2.997 ± 0.091
Flavonols	Kaempferol-3-O-rutinoside	0.089 ± 0.011
Flavonols	Myricetin	8.137 ± 0.141
Flavonols	Quercetin	66.402 ± 1.131
Flavonols	Quercetin-3-Glc-Ara	0.065 ± 0.021
Flavonols	Quercetin-3-O-glucuronide	0.078 ± 0.021
Flavonols	Quercetin-3-O-rhamnoside	0.305 ± 0.041
Flavonols	Rhamnetin	0.150 ± 0.071
Flavonols	Rutin	1.326 ± 0.081
Flavonols	Syringetin	1.811 ± 0.051
Flavonols	Syringetin-3-O-glucoside	0.023 ± 0.011
Hydroquinone derivative	Arbutin	1.279 ± 0.071

**Table 2:** Physicochemical properties of nanostructured lipid carrier (NLC) formulations, including polydispersity index (PDI), hydrodynamic diameter (HD), zeta potential (ZP), total phenolic content (TP), and encapsulation efficiency (EE%).

Formulation	PDI	HD (nm)	ZP (mV)	TP (mg GAE/mL NLC)	EE%
NLC-Blank	0.14 ± 0.02	46.71 ± 0.20	−19.39 ± 0.76	-	-
NLC-NaDES	0.26 ± 0.02	196.73 ± 2.37	−2.83 ± 0.27	-	-
NLC-TAP	0.23 ± 0.01	199.13 ± 0.91	−4.31 ± 0.16	27.05 ± 0.97	83.95
NLC-TAP-NaDES	0.21 ± 0.03	159.00 ± 0.66	−2.35 ± 0.35	20.64 ± 0.58	85.81

## Data Availability

Data are contained within the article.
